# An unusual collateral from the sinoatrial nodal artery to the left carotid artery in a patient with Takayasu arteritis: a case report

**DOI:** 10.1186/s13256-023-03752-0

**Published:** 2023-01-21

**Authors:** Marcel A. Beijk, R. Nils Planken

**Affiliations:** 1 Department of Cardiology, Amsterdam University Medical Center, University of Amsterdam, Meibergdreef 9, 1105 AZ Amsterdam, The Netherlands; 2 Department of Radiology and Nuclear Medicine, Amsterdam University Medical Center, University of Amsterdam, Meibergdreef 9, 1105 AZ Amsterdam, The Netherlands

**Keywords:** Takayasu arteritis, Coronary angiography, Collateral, Sinoatrial nodal artery, Case report

## Abstract

**Background:**

Takayasu arteritis is a noninfective chronic vasculitis mainly involving the aorta and its main branches. The presentation of the disease is heterogeneous, ranging from asymptomatic to catastrophic illness.

**Case presentation:**

A 52-year-old Caucasian woman with known Takayasu arteritis was referred to our hospital as she suffered symptoms suspicious for cerebral hypoperfusion. Computed tomography thorax angiography revealed occlusion of all aortic arch arteries and previous surgical bypasses. Cerebral perfusion depended on a few collaterals (from the distal common carotid artery to the right internal carotid artery and from the left and right internal mammary artery and the dorsal thoracic branches to both vertebral arteries). In addition, preoperative coronary angiography revealed an unusual and large collateral arising from the sinoatrial nodal artery to the left carotid artery.

**Conclusion:**

Takayasu arteritis is a relatively rare disease, with various and sometimes devastating neurological manifestations due to occlusion of the aortic arch branches. Our case highlights the importance of a thorough preoperative evaluation in search of collateral supply in symptomatic patients with Takayasu arteritis.

**Supplementary Information:**

The online version contains supplementary material available at 10.1186/s13256-023-03752-0.

## Background

Takayasu arteritis (TA) is a noninfective granulomatous vasculitis of large- and medium-sized arteries with a predilection for the aorta and its major branches. Lesions produced by the inflammatory process can either be stenotic, occlusive, or aneurysmatic. In TA there are three stages: (a) early systemic stage (prevasculitic) with constitutional symptoms; (b) vascular inflammatory stage with occurrence of stenoses, aneurysms, and vascular pain leading to symptoms; and (c) burned-out stage with development of fibrosis and collaterals; this stage is generally associated with remission. The presentation of TA is heterogeneous and most patients present late, delaying the time of diagnosis and installment of treatment. Approximately 10% of patients with TA are asymptomatic and the disease may be detected based on abnormal vascular findings on examination [1]. We report a case of a 52-year-old woman with known TA who suffered symptoms suspicious for cerebral hypoperfusion due to occlusion of the aortic arch branches. Cerebral perfusion was depending from several collaterals including an unusual collateral arising from the sinoatrial nodal artery to the left carotid artery. To our knowledge, such a collateral arising from the sinoatrial nodal artery has not been reported previously and emphasizes the importance of a thorough preoperative evaluation in search of collateral supply in symptomatic patients with Takayasu arteritis.

## Case report

**Fig. 1:**
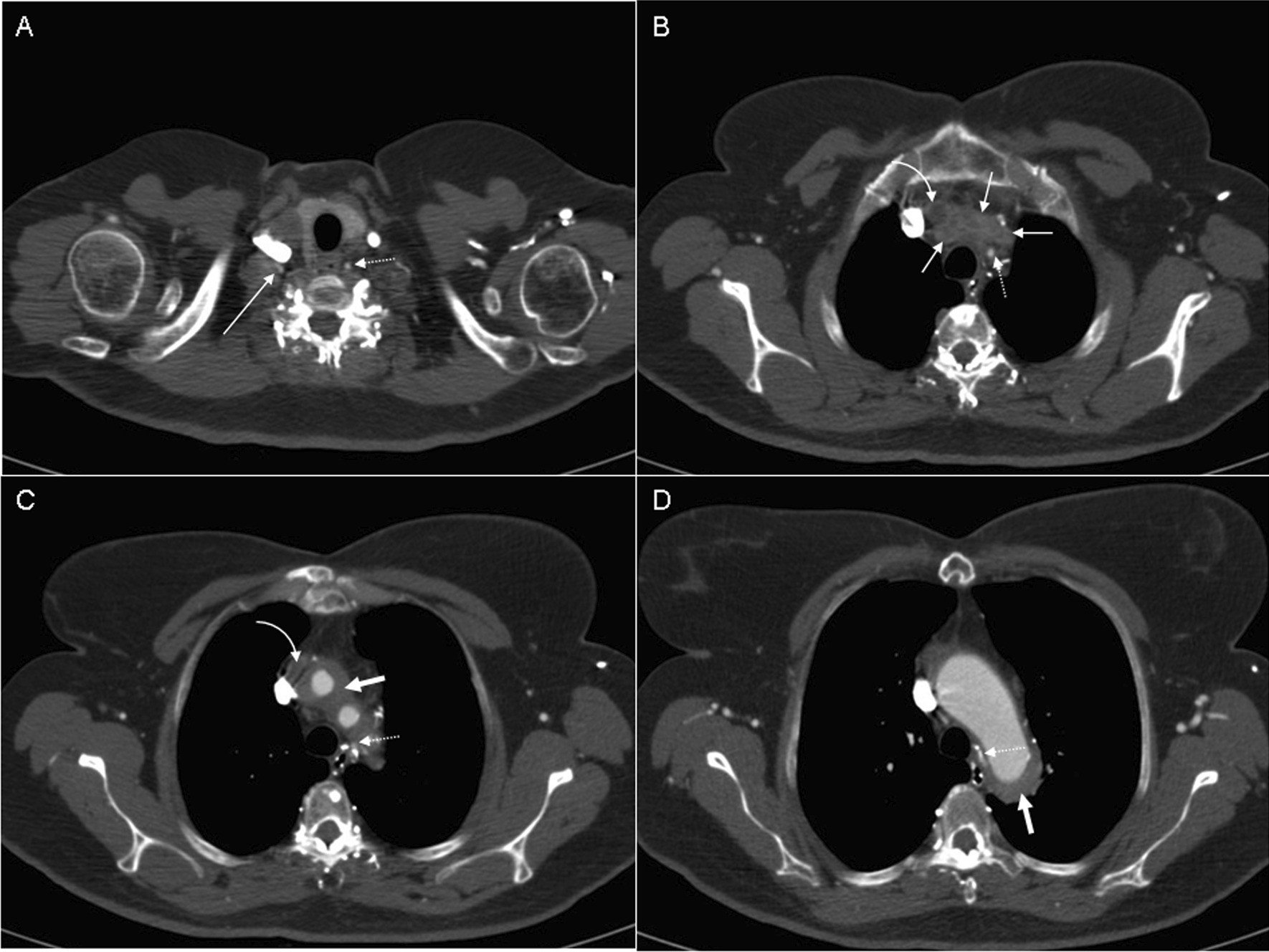
Selected axial CTA images (64 slice CT scanner) in craniocaudal order. Thickened vessel wall and infiltration (thick arrow **C**, **D**) surrounding the aortic arch and occluded supra-aortic vessels (thin arrows **B**) and occluded aorto-carotid bypass (curved arrow **B**, **C**). The collateral artery (dotted arrow **A**–**D**) arising from the right coronary artery is connected to the left vertebral artery that is one of the few vessels delivering cerebral blood supply. An additional collateral artery arising from the intercostal arteries feeds the right subclavian artery and thereby the right vertebral artery (long thin arrow **A**). Both subclavian arteries depend on collateral blood supply derived from intercostal collaterals

**Fig. 2:**
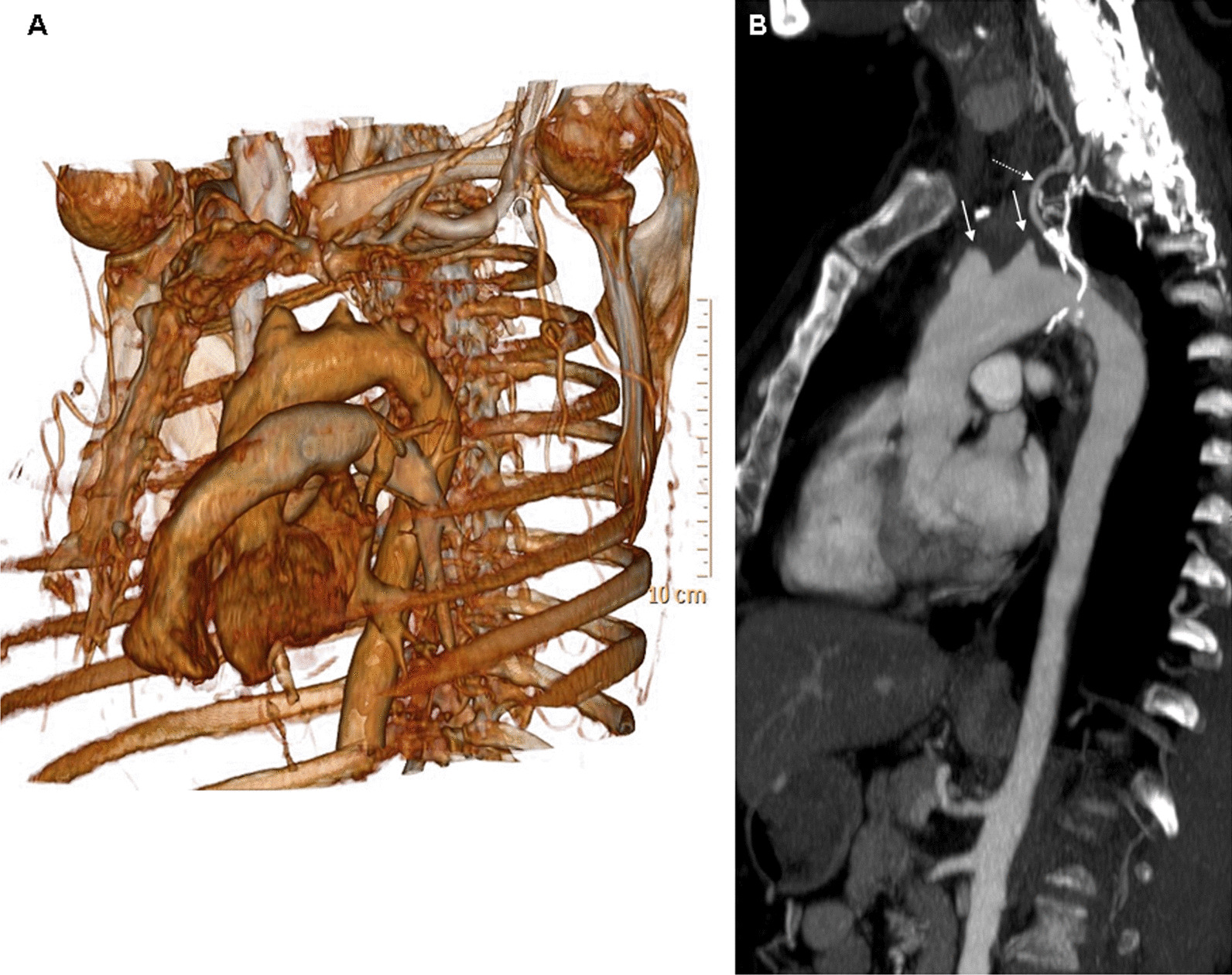
Volume-rendered CTA image, left anterior oblique orientation depicting the supra-aortic vessels occlusion (**A**). Curved multiplane reformat displaying the occluded supra-aortic vessels (arrows) and the collateral artery (dotted arrow) arising from the right coronary artery feeding the left vertebral artery (**B**)

A 52-year-old Caucasian woman was admitted to the emergency department because of an altered mental status, progressive headache, dizziness, and blurred vision since 1 week. Notably, her symptoms diminished while laying down. She was known to have TA complicated by several stenoses of the ostia of the aortic arch branches, for which she had undergone bilateral bypass surgery from the ascending aorta to the subclavian artery and carotid artery in 1998. As all anastomoses were occluded in 1999, she underwent surgical revision and a bypass from the ascending aorta to the right carotid artery was constructed. Recently, she had suffered recurrent transient ischemic attacks. Moreover, she was known to have insulin-dependent diabetes mellitus complicated by mild retinopathy and nephropathy, hyperlipidaemia, a previous cerebrovascular accident, and epilepsy. Despite of TA, she was still a heavy smoker. Physical examination revealed a blood pressure of 83/66 mmHg (measured at the leg as she had no pulsations at both arms) and a pulse of 109 bpm. Neurological examination revealed an already known mild hemiparesis of the left arm and leg, and hyperreflexia on the left side. Computed tomography (CT) of cerebrum was performed, which showed multiple demarcated hypodense regions in both cerebral hemispheres as a consequence of previous strokes without signs of recent ischemia. Under suspicion of bypass graft failure resulting in cerebral hypoperfusion, the patient was admitted. During admission she suffered from recurrent syncope with a notable improvement of symptoms in the recumbent position. A CT thorax angiography was performed showing (a) thickening of the aortic arch and proximal brachiocephalic arteries; (b) occlusion of the brachiocephalic arteries, common carotid artery, left internal carotid artery, and bypasses; (c) perfusion of the distal common carotid artery via collaterals with open right internal carotid artery; and d) both vertebral arteries perfused via collaterals from the left and right internal mammary artery and collaterals from the dorsal thoracic branches (Figs. 1 and 2). After multidisciplinary discussion, the patient was scheduled for revascularization of the internal carotid artery.

Preoperative cardiac evaluation showed a normal electrocardiogram (ECG). Echocardiography revealed a normal left and right ventricular systolic function and absence of valve disease. Finally, coronary angiography revealed absence of coronary artery disease. However, a remarkable large collateral artery was present originating from the sinoatrial nodal artery of a dominant right coronary artery that formed a bypass to the left carotid artery (Additional file 1: Video S1, Additional file 2: Video S2, Additional file 3: Video S3).

The patient underwent an uncomplicated operation under perioperative electroencephalographic surveillance with placement of a Gore-tex bypass from the ascending aorta to the right internal carotid artery. Immediately postoperative, her symptoms improved. After discharge, the patient remained a heavy smoker and had poorly regulated diabetes mellitus. Yet she remained without neurologic symptoms until 4 years after surgery when she suffered a cerebrovascular accident of the right middle cerebral artery due to a stenosis of the aorta–carotid bypass. As there were no signs of TA activity, the patient underwent successful surgical revision of the bypass.

## Discussion and conclusions

TA is a relatively rare chronic inflammatory and stenotic disease that mainly affects the aorta and its main branches (the brachiocephalic, carotid, subclavian, vertebral, and renal arteries), although the coronary and pulmonary arteries can be affected as well. A worldwide incidence of 0.4–2.6 cases per million annually has been reported. However, it is observed more frequently in Asian countries and Central and South America. Approximately 80% of patients with TA are female and the mean age of onset is approximately 30 years [2].

Although the etiology is unknown, the underlying pathologic process of TA is inflammatory. It is associated with an inflammatory cellular infiltrate in the aortic media, adventitia, and vasa vasorum that consists predominantly of lymphocytes, macrophages, and multinucleated giant cells. Advanced lesions demonstrate a panarteriitis with intimal proliferation. Over time, scarring of the aortic wall and destruction of the elastic lamina occurs.

As laboratory tests are nonspecific for TA, imaging studies such as CT scanning and magnetic resonance imaging may show typical patterns of stenoses or aneurysms of the arteries. However, angiography remains the cornerstone for diagnosis and evaluation of the extent of disease. TA can be divided into the following six types based on angiographic involvement: (a) Type I—branches of the aortic; (b) Type IIa—ascending aorta, aortic arch, and its branches; (c) Type IIb—Type IIa region plus thoracic descending aorta; (d) Type III—thoracic descending aorta, abdominal aorta, renal arteries, or a combination; (e) Type IV—abdominal aorta, renal arteries, or both; and (f) Type V—entire aorta and its branches [3]. Tissue biopsy does not play an important role in the diagnosis of TA, as histologic examination of the great vessels is usually possible only at the time of vascular surgery or postmortem.

Our patient had stenoses of all aortic arch arteries and previous surgical bypasses, with the coronary arteries unaffected (Type I). Cerebral perfusion was being maintained by the collateral circulation including an unusual and large collateral from the sinoatrial artery. Development of a collateral circulation can be seen in TA’s third stage of quiescence due to the chronic nature of the illness. Moreover, neurological manifestations can be observed in about 20% of cases and are predominantly ischemic in nature due to stenoses of the vessels. A CT cerebrum in our patient showed multiple demarcated hypodense regions in both cerebral hemispheres as a consequence of previous strokes but without signs of recent ischemia. The episodic (postural) neurological dysfunction likely caused by cerebral hypoperfusion eventually led to recurrent (near)-syncope. In general, complications of TA may include the following: cerebrovascular accidents, seizures, ischemia, organ failure, complications of hypertension, or graft stenoses and/or occlusion. In patients with two or more complications, the 5- and 10-year survival rates are estimated to be 69% and 36%, respectively. In patients with one or no complication, it is estimated to be 100% and 96%, respectively [1, 4]. Strict management of traditional cardiovascular risk factors is mandatory to minimize secondary cardiovascular complications. Our patients has had several vascular complications, multiple strokes, seizures, and graft occlusions. She remained a heavy smoker as multiple attempts to quit smoking failed, which may have contributed to the failure of the aorta–carotid graft. In the active stage of TA, glucocorticoid treatment aims to halt the inflammation and prevent development of stenoses in the arteries. When the response to steroids is inadequate, cytotoxic agents such as methotrexate, azathioprine, and cyclophosphamide can be used. Moreover, anti-tumor necrosis factor (TNF) agents have shown encouraging results but further evaluation is needed. Critical stenotic lesions can be treated by bypass graft surgery providing good long-term patency rate. Percutaneous balloon angioplasty may serve as an alternative in case of short lesions. The use of conventional stents seems to be associated with high failure rates in patients with TA.

## Conclusion

TA is a relatively rare disease with a heterogeneous presentation with neurological manifestations in the minority of cases. The third stage of TA is characterized by grow of collaterals to bypass stenotic and occluded vessels. We present this case to highlight that rare and unusual collaterals may be formed, and to emphasize the importance of a detailed angiographic evaluation to discover the presence of these rare collateral formations.

## Supplementary Information

**Additional file 1: Video S1.** LAD. Left coronary artery in the RAO view.

**Additional file 2: Video S2.** RCA. Right coronary artery in the RAO view

**Additional file 3: Video S3.** RCA-anastomosis. Right coronary artery and an unusual collateral form the sinoatrial nodal artery to the left vertebral artery in the AP view

## Data Availability

Not available.

## Abbreviations

TA: Takayasu arteritis
CT: Computed tomography
ECG: Electrocardiogram
